# An Improved Simulated Annealing Technique for Enhanced Mobility in Smart Cities

**DOI:** 10.3390/s16071013

**Published:** 2016-06-30

**Authors:** Hayder Amer, Naveed Salman, Matthew Hawes, Moumena Chaqfeh, Lyudmila Mihaylova, Martin Mayfield

**Affiliations:** 1 Department of Automatic Control and Systems Engineering, University of Sheffield, Sheffield S1 3JD, UK; n.salman@sheffield.ac.uk (N.S.); m.hawes@sheffield.ac.uk (M.H.); l.s.mihaylova@sheffield.ac.uk (L.M.); 2 College of Information Technology, UAE University, Al Ain 15551, United Arab Emirates; moumena@uaeu.ac.ae; 3 Department of Civil and Structural Engineering, University of Sheffield, Sheffield S1 3JD, UK; martin.mayfield@sheffield.ac.uk

**Keywords:** traffic congestion, Internet of Vehicles, Internet of Things, simulated annealing, multi-objective optimisation, vehicle re-routing

## Abstract

Vehicular traffic congestion is a significant problem that arises in many cities. This is due to the increasing number of vehicles that are driving on city roads of limited capacity. The vehicular congestion significantly impacts travel distance, travel time, fuel consumption and air pollution. Avoidance of traffic congestion and providing drivers with optimal paths are not trivial tasks. The key contribution of this work consists of the developed approach for dynamic calculation of optimal traffic routes. Two attributes (the average travel speed of the traffic and the roads’ length) are utilized by the proposed method to find the optimal paths. The average travel speed values can be obtained from the sensors deployed in smart cities and communicated to vehicles via the Internet of Vehicles and roadside communication units. The performance of the proposed algorithm is compared to three other algorithms: the simulated annealing weighted sum, the simulated annealing technique for order preference by similarity to the ideal solution and the Dijkstra algorithm. The weighted sum and technique for order preference by similarity to the ideal solution methods are used to formulate different attributes in the simulated annealing cost function. According to the Sheffield scenario, simulation results show that the improved simulated annealing technique for order preference by similarity to the ideal solution method improves the traffic performance in the presence of congestion by an overall average of 19.22% in terms of travel time, fuel consumption and *CO*_2_ emissions as compared to other algorithms; also, similar performance patterns were achieved for the Birmingham test scenario.

## 1. Introduction

The explosive growth of the global economy has led to an expansion of cities. There has been an increase in the population mass and, therefore, an increase in the number of vehicles driving on city road networks of limited capacity. This has prompted an extreme increase of traffic congestion, road accidents and air pollution. Some studies have revealed that 30% of *CO*_2_ emissions are due to inefficient vehicle management [1]. This has resulted in significant economic and productivity losses, making improvement of mobility a key challenge within smart cities. Solving such a problem can be aided by information obtained via sensors deployed as part of smart city initiatives, which then have to be communicated to vehicles/drivers to allow them to make a decision with regards to alternative routes. Once such a decision had been made, it would also be feasible to eventually see information regarding planned routes to be communicated back from the vehicles to the smart city infrastructure. Such information can then be used to predict the number of vehicles at each intersection within a smart city, which in turn could be used to adapt the sequences of traffic lights to allow a more overall optimal traffic flow for the city as a whole. A further example of how this can be used within smart cities would be related to reducing the concentrations of air pollution within the city. This would be achieved by redirecting heavily polluting vehicles away from areas with high pollution levels.

As a result, there has been a significant body of research dealing with the algorithms to reduce traffic jams. The Dijkstra [2] and the A* algorithms [3] are the two most common path planning algorithms. Other studies concentrate on the integration of swarm intelligence algorithms, such as artificial ant colony algorithms [4], genetic and simulated annealing algorithms [5]. However, most of the recently developed algorithms are generally designed for static graphs. They are not designed to be employed for dynamic path planning in a real-time traffic environment. This is due to the unpredictable traffic conditions that might occur in the dynamic environment.

Recently, hybrid systems have been suggested to integrate algorithms in each group to create a vigorous integrated solution to overcome any drawbacks or obstructions on each single approach; for example, the hybrid genetic algorithm using Dijkstra’s heuristic multi-objective optimization for dynamic route planning with the predicted traffic in a real-world road network [6].

There is a significant increase in developing efficient solutions for improved mobility in intelligent transportation systems (ITS) [7]. Finding the optimal navigation route, from a source to a destination, within a reasonable time is the key task. Different cost functions can be applied, such as attaining the minimum travel distance (TD), minimum travel time (TT) or minimum fuel consumption. ITS is comprised of a broad range of wireless communication-based information, control and technologies. When combined with the transportation system infrastructure and in vehicles themselves, this technology helps to manage traffic flow, reduce traffic jams, enhance road safety, provide alternative routes to commuters, reduce fuel consumption, time, money and vehicle emissions in congested urban areas [8].

An efficient approach can be developed with the aid of the Internet of Things (IoT). The IoT is an envisaged communication system that allows various devices, such as wireless sensor networks (WSN), mobile phones, mobile phone masts, actuators and near-field communication devices (NFC), to communicate and collaborate with each other to complete a common goal [9]. The Internet of Vehicles (IoV), a natural offshoot of the IoT, foresees all future vehicles to be connected, sharing information related to safety, convenience and infotainment. Vehicle to vehicle (V2V) and vehicle to infrastructure (V2I) communication systems are two subset of IoV. The V2V communication system allows two vehicles to interact directly without relying on fixed infrastructure. On the contrary, the V2I communication system allows vehicles to communicate with roadside units (RSU), such as sensors, traffic light controllers, intelligent signals, mobile phone masts, etc., which are installed as part of the smart city concept [10]. V2V and V2I have been widely utilized to resolve various complications related to transportation in smart cities, in which the most significant problem is the traffic congestion.

IoV systems can significantly improve traffic safety and convenience in smart cities, by providing drivers with timely information about road conditions and travel situations. Existing solutions to the traffic congestion problem by utilizing vehicular communication rely either on V2V communication alone or both V2V and V2I communications. However, there are still some Limitations, which can be summarized as follows:

1. A large portion of the previous or current vehicle routing algorithms attempt to identify the minimum TD or TT. Generally, they cannot attain an active trade-off.
2. Utilizing just individual traffic information or a single cost function for the vehicle routing problem is not satisfactory. Different navigation criteria should be considered to find the optimal path of the driver. This will help drivers to have different navigation options, which can be the fastest route, the least congested, the least fuel consumption and the least air pollution.

This paper presents and evaluates a new multi-objective improved simulated annealing technique for order preference by similarity to the ideal solution (ISATOPSIS) algorithm for congestion avoidance based on an IoV communication system. The main objective in ISATOPSIS is to provide various route decisions according to different objectives in order to meet the diverse navigation requirements of drivers; for example, the minimum TT, TD, fuel consumption or a trade-off of all conditions. In this paper, two other algorithms have been implemented: simulated annealing weighted sum (SAWS) and the simulated annealing technique for order preference by similarity to the ideal solution (SATOPSIS) for compression purposes. The cost function of SAWS has been formulated using the weighted sum method. The cost function of SATOPSIS and ISATOPSIS has been formulated using the multi-attribute decision making (MADM) method, which is called the technique for order preference by similarity to the ideal solution (TOPSIS) method [11]. The results of the proposed algorithm ISATOPSIS have been compared to the shortest path Dijkstra algorithm (DA), SAWS and SATOPSIS.

The remainder of the paper is organized as follows: In Section 2, a literature review of related work is presented. The details of SAWS, SATOPSIS and ISATOPSIS are given in Section 3. In Section 4, a performance evaluation is provided. Finally, conclusions are drawn in Section 5.

## 2. Related Work

Numerous studies have been published on IoV as part of IoT to find a solution to the traffic congestion problem in order to reduce the TD, TT and fuel consumption. In this section, a discussion and review of these routing algorithms is given.

In [12], the algorithm aims to send geo-broadcast messages in V2V and V2I communications. The algorithm used an event-driven message instead of a beacon message sent periodically. This message is transmitted to all vehicles that are within a specific range to provide the driver with information about traffic conditions. However, this approach focuses only on providing information to other vehicles without any mechanism to avoid the congested area.

In [13], the authors employ V2V communication with messages periodically sent between vehicles. They propose a technique called content-oriented communication (COC) to solve traffic congestion. With this method, the vehicle evaluates the road traffic density from messages received from other vehicles and periodically sends these data to the furthest vehicles within communication range. As a result, vehicles obtain traffic density information for different locations and detect congestion levels by comparing the road traffic density calculated with average traffic density values for the road segment. However, this work did not propose a mechanism to avoid traffic jams.

In [14], a new Dijkstra algorithm was proposed. This algorithm encompasses new inputs for vehicle path planning, along with a reactive planner to update the selected path according to the required route changes. A general classification of vehicle routing algorithms and evaluation metrics in smart cities is also presented. However, this is done without any performance evaluation.

In [15], the authors evaluate four algorithms: static Dijkstra, static A*, dynamic Dijkstra and dynamic A*. The evaluated algorithms have been applied in three different test scenarios (city centre, suburban and rural). According to the performance evaluation, the authors recommend the A*-based algorithms as the best routing algorithms in the differing road environments. However, the drawback of these algorithms is that they utilize only a single objective when finding the alternative paths, which leads to the transfer of the congestion to other roads.

In [16], the authors propose an algorithm called cooperative traffic congestion detection (CoTEC), which uses fuzzy logic control with two inputs (the speed and the density of vehicles) and one output (congestion level). This algorithm uses cooperative awareness messages (CAM) or beacon messages, sent periodically to other vehicles telling them to avoid the congested area. In addition, it detects the congestion by using an external metric called the level of service (LOS) developed by Skycomp. This involves the classification of congestion levels from aerial surveys of highways. Once obtained, this information is then combined with data from organizations, such as local authorities. As a result, the performance of the algorithm is heavily dependent on the accuracy of the data from these external organizations. Additionally, no mechanism is provided for drivers to find the optimal route for their journey.

In [17], a centralized framework is proposed to obtain real-time coordinates of vehicles and their direction and velocity, which can be used to determine traffic congestion levels. When congestion is detected, vehicles close by are re-routed using two algorithms. The first algorithm, dynamic shortest path (DSP), allocates to each vehicle the shortest path (smallest TT) to reach their destination. However, the drawback of the first algorithm is the probability of transferring the congestion to other areas. Therefore, the second algorithm, random multipath *K* shortest paths (RKSP), determines the *K* shortest paths for each vehicle and randomly allocates the vehicle to one of them. As a result of using *K* alternative paths, it is possible to ensure that the congestion is not just transferred to a different road. However, the drawback of this algorithm is that the choice of routes is arbitrary and uses a single objective function.

In [18], the authors propose an ant-based congestion avoidance system (AVCAS). AVCAS integrates the average travel speed prediction with segmentation of a city map to detect and reduce the congestion. It collects the traffic information from V2V communications and RSU to forecast the average speed of the roads. Then, it uses a weighted sum method to formulate the cost function for the roads, where the weights have been calculated by trial and error. However, this algorithm still chooses the shortest paths when re-routing the vehicles to avoid the congestion. As a result, this transfers the congestion to a new road.

In [19], the authors describe a distributed real-time V2V congestion evasion technique. Congestion levels are detected and vehicles re-routed using congestion messages called request/receive messages (instead of beacon messages). When a vehicle reaches an intersection and can select from different paths, it sends a congestion request packet to all neighbouring vehicles within its communication range to obtain the traffic status information. This message encompasses a list of roads that form potential paths to their destination. The vehicle will select its next potential route based on the received traffic data. A congestion response message is transmitted only when a congestion request is received. If the congestion request message is received twice, the vehicle will not send a response message, and the congestion message will be discarded from the database of the receiving vehicle. This ensures that no redundant information is transmitted. Otherwise, the vehicle will broadcast the response message, which includes data about the congested roads available at the receiver. The vehicle that receives the congestion message request will select the road that has the minimum travel time. However, this method has two drawbacks. Firstly, it includes a high delay time due to sending the traffic data. Secondly, the authors have not considered the high probability and consequences of lost packets in a built up area.

In [20], the authors suggest an ITS based on RSUs in order to avoid traffic congestion. This system is triggered only when an accident occurs. The vehicles involved start to transmit alert messages with the location of the accident to the other vehicles within their transmission range. Every vehicle that has received an alert message checks if it is near an affected area. If it is far from the accident point, the vehicle will delete the received message. However, if the vehicle is near the accident point, it checks its route to establish whether it is affected by the accident or not. At this stage, a new route can be calculated and assigned to the vehicle. However, the drawback of this method is that the re-routing is done based on either a static DA or A* algorithm. This would transfer the congestion from one area to another. In addition, this system does not consider the traffic congestion that occurs due to the high vehicle densities in a given area.

Recently, in [21,22], the authors proposed two systems called geographical accident aware of reducing urban congestion (GARUDA) and a solution using cooperative re-routing to prevent congestion and improve traffic condition (SCORPION) based on the ITS and IoV. The GARUDA is a decentralized system, and it offers a new path to the drivers considering all cars and available routes in the region nearest to an accident. The SCORPION is a centralized system that collects the traffic data from RSU, and it uses the concept of fuzzy logic to predict congested roads. However, both systems propose the re-routing based on static Dijkstra or A* algorithms and, thus, will have the same shortcoming as in [15], i.e., the possibility of moving congestion to a new area.

Other studies have reviewed the most relevant algorithms to calculate the route in the vehicle route problem (VRP), such as in [2,3,4,6] and [23]. In [23], a modified version of ACO is proposed in order to reduce the travel time for vehicles on the move. The modified ant colony optimization (MACO) is a variation of the classical ACO in which the idea of an ant colony has been reversed. Instead of attracting the vehicles toward roads that have high pheromone levels, the MACO algorithm routes and disperses the traffic toward paths with lower pheromone values to avoid the congestion. However, the cost function of this algorithm is very similar to the classical Dijkstra algorithm.

Hence, the novelty of this work is the developed dynamic multi-objective optimization algorithm, which combines simulated annealing (SA) with the MADM TOPSIS cost function to provide the driver with optimal paths. In addition, the proposed algorithm utilizes real-time data using IoV, unlike other works, where the statical DA or A* is used to re-route the vehicles and leads to the transfer of congestion onto other roads. Our proposed approach is also better than the ACO-based methods as the ACO can become stuck in local optima solutions (rather than the global solution). This is because of the ACO updating the pheromone based on the current optimal route [24].

The proposed algorithm has four features:

1. ISATOPSIS allows transition from a good solution to a worse solution under a strict condition. This allows the algorithm to find the global optimal solution and avoid becoming stuck in local optimal solutions.
2. ISATOPSIS can work for dynamic path planning by collecting real-time traffic data from IoV and efficiently finding alternative routes for the driver.
3. ISATOPSIS can optimize more than one criteria using the MADM TOPSIS method, which allows alternative routes to be judged on different criteria.
4. ISATOPSIS periodically detects and avoids congestion by selecting the paths that have the minimum traffic, *CO*_2_ emissions, fuel consumption, as well as travel time. This is due to combining different navigation attributes in the cost function.

## 3. System Description

IoV consists of two components: V2V and V2I communication. The V2V system is an on-board WSN, which is installed in the vehicles themselves. The sensors allow the vehicles to send and receive information, such as speed, location and direction [10]. The V2I system is a roadside unit network comprising some of the infrastructure related to smart cities, e.g., traffic sensors deployed along the roads and at intersections.

**Figure 1:**
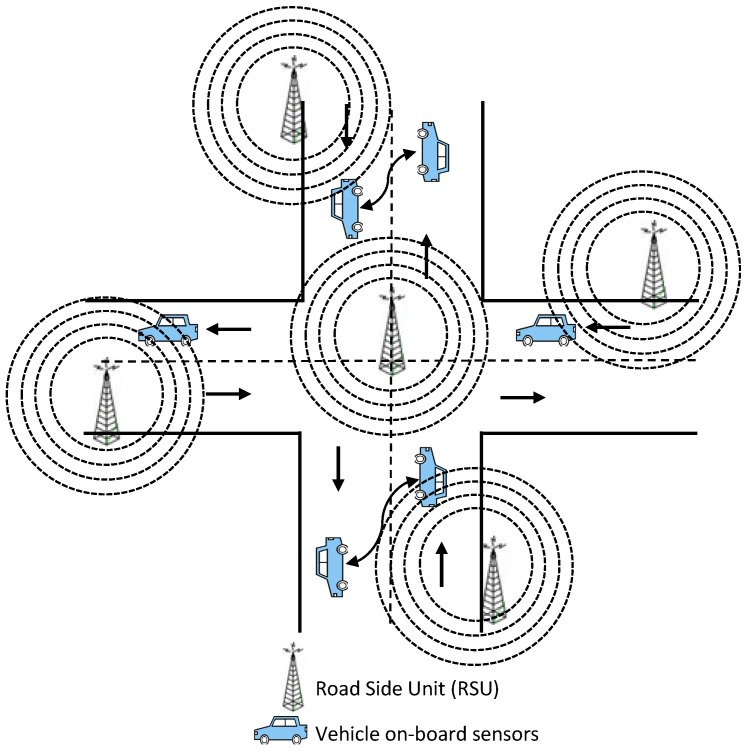
Internet of Vehicles (IoV) road network infrastructure.

In this paper, both systems have been used with the “hello” protocol [25] or beacon messages, which focus on tackling the problem of monitoring, traffic and reducing congestion. Figure 1 shows the V2V and V2I architectures. In this protocol, each vehicle will have an overview of the average speed and density of vehicles on the road network. This allows them to choose the optimal path to reach their destination. In this section, we describe our proposed system by specifying the data dissemination methodology, the road network model, simulated annealing of SAWS and SATOPSIS and an improved simulated annealing TOPSIS algorithm.

### 3.1. Data Dissemination

The data have been transmitted using beacon messages, and the proposed protocol works as follows: the vehicles transmit their average velocity and “roadId” to their neighbouring RSUs through beacon messages. Each RSU holds a data structure containing the average speeds, roadIds and roads length of all vehicles within its transmission range. The average speeds are found from the speed measurements over the previous five seconds (one measurement a second). If the average speed is less than or equal to a velocity threshold predetermined by the designer, the congestion detection is initiated, in which congested roads are identified. The RSU will then verify whether or not to broadcast the data from receiving beacon messages. The data will be used if the RSU does not receive a duplicate message with the same roadId. Whenever the RSU receives a new beacon, it updates its data structure and broadcasts the data to vehicles within its transmission range. As a result, congested roads can be excluded from the map and a new route calculated at the application layer of V2V communication using the ISATOPSIS algorithm.

### 3.2. Road Network

The road network can be modelled as a directed graph G=(N,E) , where *N* corresponds to the intersections (nodes) and $E=\{e_{1},e_{2},\cdots ,e_{i}\}$ corresponds to the road segments (edges). The road matrix *A* can be formulated as follows: suppose each intersection contains *n* roads; each of the roads contains the *j* attribute value in the road network:

(1) $$A=\begin{array}{l} A_{1}\\ A_{2}\\ \vdots \\ A_{n} \end{array}\begin{array}{c} \begin{array}{cc} C_{L} & C_{S} \end{array}\\ \left[\begin{array}{cc} r_{11} & r_{12}\\ r_{21} & r_{22}\\ \vdots & \vdots \\ r_{n1} & r_{n2}\\ w_{1} & w_{2} \end{array}\right] \end{array}$$

The normalized road matrix has been obtained using the following equation:

(2) $$r_{kj}=\frac{x_{kj}}{\sqrt{\sum_{k=1}^{n}{\left(x_{kj}\right)}^{2}}}\;\text{where}\;k=1,\cdots ,n;\;j=1,2$$

where $r=\{r_{kj}|\;k=1,\cdots ,n;\;j=1,2\}$ are the normalized performance values of each $C_{L}$ and $C_{S}$ , respectively. $X=\{x_{kj}|\;k=1,\cdots ,n;\;j=1,2\}$ denotes the set of performance values of each $C_{L}$ and $C_{S}$ , respectively. $w=\{w_{j}|j=1,2\}$ denotes the set of weights; $V=\{v_{1},v_{2},\cdots ,v_{j}\}$ is the set of vehicles; and $A=\{A_{k}|\;k=1,\cdots ,n\}$ are the alternative roads for each vehicle in *V*. Every vehicle *v_j_* periodically sends a message *msg_j_* that contains {*roadId_j_*, *averagespeed_j_*, *position_j_*, *route_j_*, *destination_j_*} to the neighbouring RSUs.

Two parameters have been used in our optimization:

1. Road length $C_{L}=\left\{r_{kj}|\;k=1,\cdots ,n;\;j=1\right\}$ represents the normalized length in a directed graph *G* for each alternative in *A*.
2. Average velocity $C_{S}=\left\{r_{kj}|\;k=1,\cdots ,n;\;j=2\right\}$ represents the normalized average speed of each vehicle at a certain period in *A*.

### 3.3. Simulated Annealing of SAWS and SATOPSIS

SA is an optimization approach first proposed by [26] that imitates the process of annealing in mineralogy. It allows transference from a given solution to a worse solution under strict conditions. This allows the algorithm a mutation to move from local minima or maxima towards the global optima.

**Figure 2:**
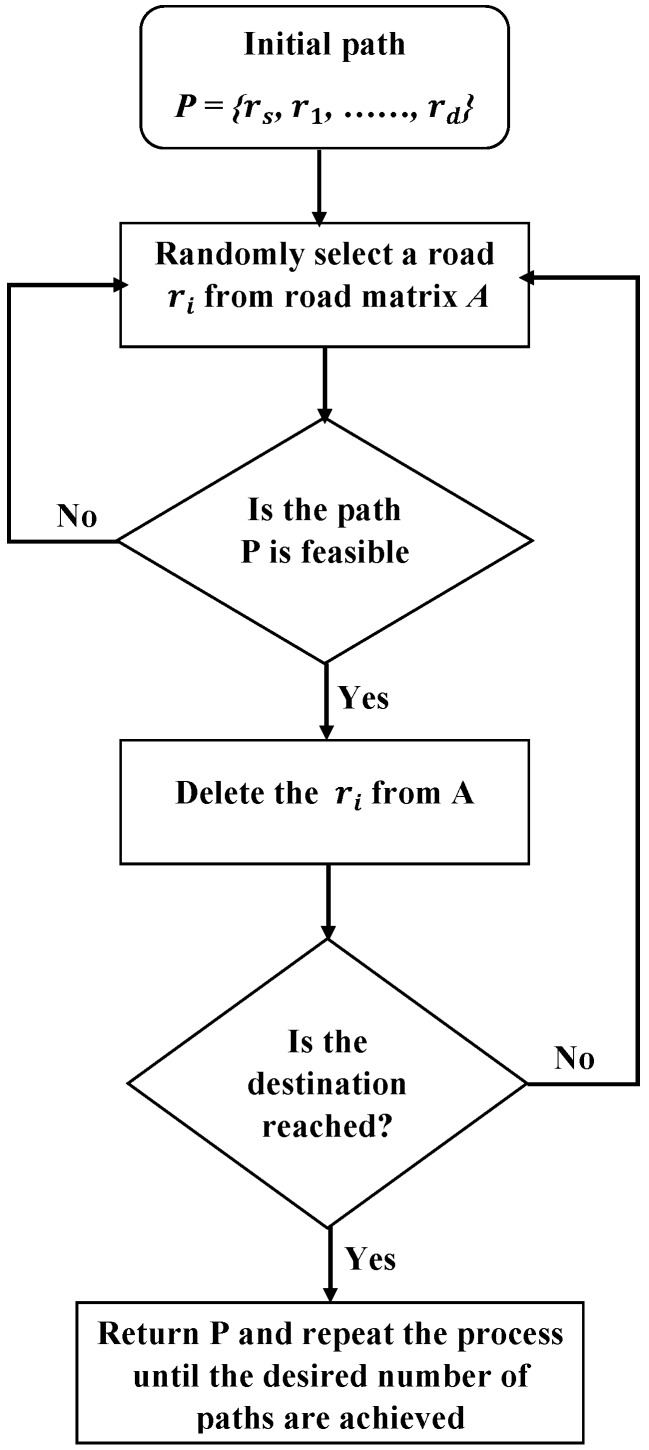
The procedure of generating a random path.

SA begins with a set of randomly-chosen solutions. Each iteration produces a new solution of the state vector, which is found based on the cost function. A new solution with a high cost is accepted immediately. However, a solution with a lower cost than the previous one can be accepted with a transition probability. In this work, *K* random paths have been generated from matrix *A*. Figure 2 shows how the paths are generated.

SA has been used with two different cost functions (the weighted sum and TOPSIS methods) to select the optimal path from the *K* random paths. Every $v_{j}$ in *V* has a set of alternative paths from $R_{k}$ , where $R_{k}$ is a matrix of *K* random paths generated from *A*. Every path in $R_{k}$ contains the number of roads $A_{k}$ , and the cost function of each path has been computed as the sum of the costs of all of the roads it contains.

Algorithm 1 describes the procedure of choosing a path from *K* random paths, where $X_{c}$ is the current solution, which is generated randomly from *A*. *T* is the temperature parameter, which is moderately decremented with time. The constant *α* is the cooling rate used to gradually decrease the value of *T*. When the temperature parameter has a very high value, i.e., T→∞ , a new path $X_{n}$ is selected randomly from $R_{k}$ . The cost function is evaluated for $X_{n}$ ( $N(X_{n})$ ) and compared to the previous value of the cost function $\left(C\left(X_{c}\right)\right)$ . If the cost function is higher than the previous value, then the solution is accepted. Even if the new solution is not appropriate (meaning the cost of the new solution is less than or equal to the current solution), it is accepted with some acceptance probability. This helps to expand the search and avoids local optima. When *T* approaches zero, paths with a high cost have a high probability of being accepted.

The SAWS and SATOPSIS find up to three alternatives for vehicles that have the same source/destination. When the optimal paths are found, they are ordered based on the cost function. The vehicles that have the same source/destination are distributed on these three optimal paths. This will ensure the vehicles are shared between the paths and ensures the traffic load is balanced on the map.
**Algorithm 1** The simulated annealing algorithm for enhancing mobility.1:
$X_{c}=X_{c_{0}}$
 Initial random solution2:
$T=T_{0}$
 An initial temperature3:*α* = Cooling rate4:
$s_{b}$
 = Current best solution5:
$s_{b}$
 ← 
$X_{c}$
6:**While**

$T>T_{m}$
 where 
$T_{m}$
 is the minimum temperature7:Generate a random neighbour solution 
$X_{n}$
 from 
$R_{k}$
8:**If**

$N(X_{n})>C(X_{c})$
9:Move to 
$X_{n}$
10:Accept change 
$s_{b}\leftarrow X_{n}$
11:**Else If**

$N(X_{n})\leq C(X_{c})$

**Then**12:Move to 
$X_{n}$
 with transition probability13:
$P_{t}=1/1+\exp\left(C\left(X_{c}\right),N\left(X_{n}\right),T\right)$
14:**Endif**15:
T=αT
16:**Endwhile** (if 
$T<T_{m}$
)17:**Return**

$s_{b}$



### 3.4. An Improved Simulated Annealing TOPSIS Algorithm

In this section, SA has been implemented for dynamic path planning using the TOPSIS cost function method. The decision-making process of the proposed approach is constructed as follows:

#### 3.4.1. Off-Line Computation of Path Planning

**Figure 3:**
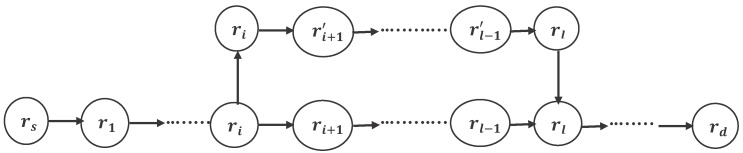
The procedure for constructing a new path *X_n_* based on an initial path *X_c_*.

SA begins with off-line route calculation, in which every $v_{j}$ in *V* has a set of roads from *A* and every road $A_{k}$ in *A* has a cost function formulated using TOPSIS method. The algorithm description of the SA is outlined in Algorithm 1, except Step 1, where $X_{c}$ is the current search solution or an initial feasible path, which is an optimal path from $R_{k}$ . When the temperature parameter has a very high value the new search solution, $X_{n}$ in Step 7 is constructed based on $X_{c}$ as follows:

1. An initial optimal path $X_{c}=\left\{r_{s},r_{1},\cdots ,r_{i},r_{i+1},\cdots ,r_{l-1},r_{l},r_{d}\right\}$ where $r_{i}$ means the *i*-th road segment.
2. The perturbation (see Figure 3) consists of the following three steps.
  (a) Two roads $r_{i}$ and $r_{l}$ , called base roads, are chosen randomly in the $X_{c}$ path.
  (b) A path is constructed, using $r_{i}$ as an origin and $r_{l}$ as a destination.
  (c) The path $X_{c}=\left\{r_{s},r_{1},\cdots ,r_{i},r_{i+1},\cdots ,r_{l-1},r_{l},r_{d}\right\}$ is replaced by ( $r_{i}$ , $\overset{´}{r_{i+1}}$ , ⋯, $\overset{´}{r_{l-1}}$ ) to give a new path $X_{n}=\left\{r_{s},r_{1},\cdots ,r_{i},\overset{´}{r_{i+1}},\cdots ,\overset{´}{r_{l-1}},r_{l},r_{d}\right\}$ .
3. Check the feasibility of the new path.
4. If its not feasible, then repeat the process. Otherwise, use SA as in Algorithm 1 and compare the cost of the new path to the previous path.

#### 3.4.2. On-Line Computation of Path Planning

**Figure 4:**
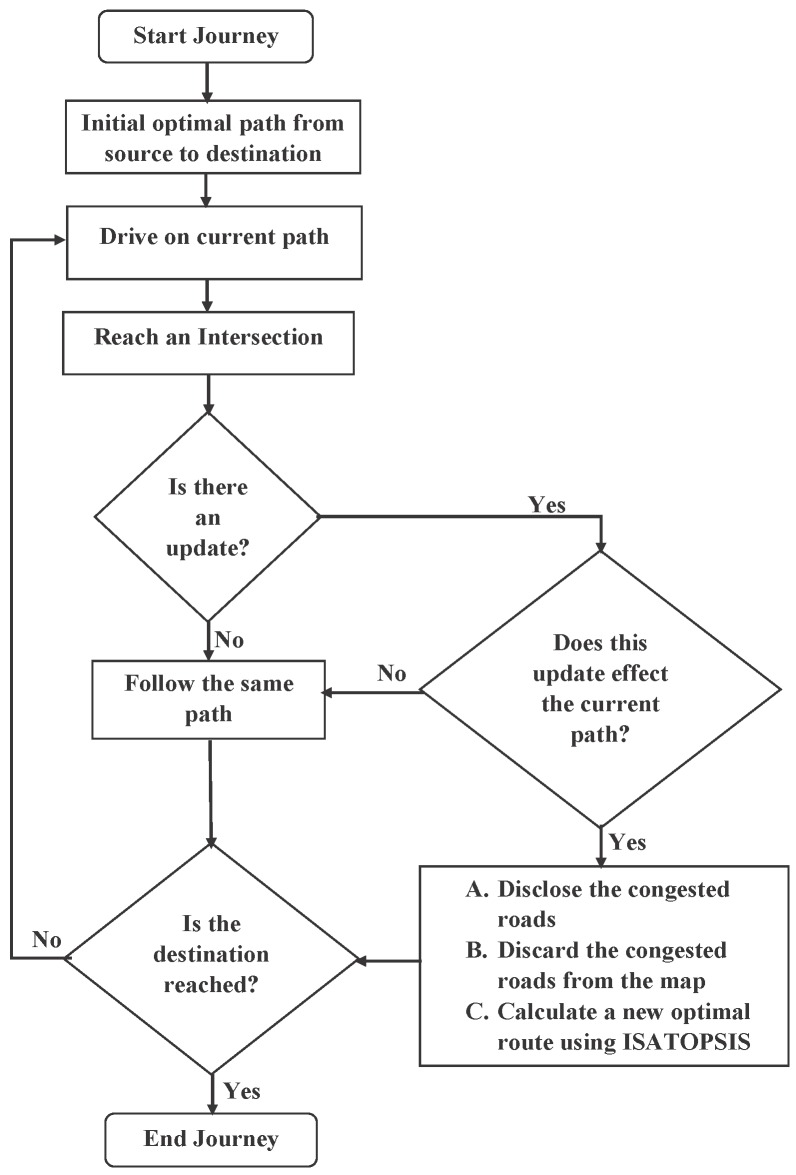
Flow chart of the simulated annealing (SA) congestion avoidance mechanism.

As discussed in the off-line computation section, the vehicles use the route generated by off-line path planning to travel through the city; the on-line path planning is then triggered to automatically compute an alternative route when congestion is detected as follows: When the vehicle reaches an intersection and enters the RSU transmission range, it will receive updated data with the table of congested roads. Then, the vehicle evaluates its current route. If the evaluation shows that the current route has not been affected by the updated data, the vehicle will keep travelling along the current route. Otherwise, if the vehicle is likely to enter a congested road, SA will be activated and reloaded with the updated search space. The updated search space contains the current status of the vehicle, its current location and the new cost of the road segments. The alternative route will be computed from the updated search space to allow the vehicle to travel from its current location to the destination. Figure 4 shows the procedure of the congestion avoidance using ISATOPSIS.

### 3.5. Calculate the Weights of SAWS, SATOPSIS and ISATOPSIS

In this paper, the standard deviation (SDV) method is used to determine the weights of multiple objectives. The weight in the MADM problem reflects the relative importance of the various objectives. The weights of the different criteria have been normalized to transform the different scales and units into common measurable units using:

(3) $$R_{kj}^{\prime }=\frac{r_{kj}-\min_{\begin{array}{c} 0\leq j\leq m \end{array}}r_{kj}}{\max_{\begin{array}{c} 0\leq j\leq m \end{array}}r_{kj}-\min_{\begin{array}{c} 0\leq j\leq m \end{array}}r_{kj}}$$

where $M^{\prime }={\left(R^{\prime }\right)}_{m\times n}$ is the matrix after range normalization and max $r_{kj}$ and min $r_{kj}$ are the maximum and the minimum values of the criterion (j) in $C_{L}$ and $C_{S}$ , respectively.

$$R_{kj}^{\prime }\in \left[0,1\right]\;k=1,\cdots ,n;\;j=1,2$$

$SDV_{j}$ is the standard deviation that is calculated independently for every *j*-th criterion using the normalized matrix $M^{\prime }={\left(R^{\prime }\right)}_{m\times n}$ as shown in (4):

(4) $$SDV_{j}=\sqrt{\frac{1}{n}\sum_{k=1}^{n}{\left(R_{kj}^{\prime }-{\bar{R}}_{j}\right)}^{2}}$$

where:

(5) $${\bar{R}}_{j}=\frac{1}{n}\sum_{k=1}^{n}R_{kj}^{\prime }$$

and ${\bar{R}}_{j}$ is the mean of the values of the *j*-th criterion in $C_{L}$ and $C_{S}$ after normalization and j=1,2 . From Equation (4), the weight $\left(w_{j}\right)$ of the criterion (*j*) in road matrix *A* can be defined as:

(6) $$w_{j}=\frac{SDV_{j}}{\sum_{j=1}^{2}SDV_{j}}$$

### 3.6. Simulated Annealing Weighted Sum Method

A multi-objective problem is often solved by combining the multiple objectives into one single-objective scalar function. This approach is in general known as the weighted-sum or scalarization method. In this paper, the cost function of the simulated annealing has been formulated using the weighted sum method as below:

(7a)f=Max{w1CL+w2CS}(7b)CL=∑k=1nrk1(7c)CS=∑k=1nrk2

### 3.7. TOPSIS Cost Function of SATOPSIS and ISATOPSIS

The SATOPSIS and ISATOPSIS cost functions have been implemented using the TOPSIS method that can determine the best alternative route based on the concepts of a compromise solution. The compromise solution can be regarded as choosing the solution with the shortest Euclidean distance from the ideal solution and the farthest Euclidean distance from the negative ideal solution. The procedures of TOPSIS can be described as follows:

1. Calculate the weighted normalized ratings by using the normalized matrix from Equations (1) and (2):
(8) $$z_{kj}=w_{j}r_{kj}$$
2. Calculate the positive and negative ideal solutions (PIS and NIS), which are the maximum and the minimum values of the criterion (*j*) in $C_{L}$ and $C_{S}$ , respectively. We can formulate the normalized road matrix and obtain the positive and negative ideal solutions as follows:
(9a)PIS=H+={z1+,⋯,zj+}(9b)NIS=H−={z1−,⋯,zj−}
3. Calculate the separation ( $D_{k}^{*}$ and $D_{k}^{-}$ ) from PIS ( $H^{+}$ ) and NIS ( $H^{-}$ ) for the alternative paths as follows:
(10a)Dk*=∑j=12(zkj−zj+)2k=1,⋯,n(10b)Dk−=∑j=12(zkj−zj−)2k=1,⋯,n
4. Calculate the cost function of SA by finding the similarities to PIS using:
(11) $$Y_{k}^{*}=\frac{D_{k}^{*}}{D_{k}^{*}+D_{k}^{-}}\;\;Y_{k}^{*}\in \left[0,1\right]\;\forall k=1,\cdots ,n$$

## 4. Performance Evaluation

This section describes the performance evaluation and the results of the proposed solution. To manage and monitor the vehicle’s mobility, we have used the Simulation for Urban Mobility (SUMO) Version 0.22.0 [27] with the Traffic Control Interface (TraCI), which is an interface between road traffic and network simulators [28]. Two scenarios have been used to test and validate the proposed algorithm (the scenarios of Sheffield city and Birmingham city).

### 4.1. Scenario of Sheffield City

**Figure 5:**
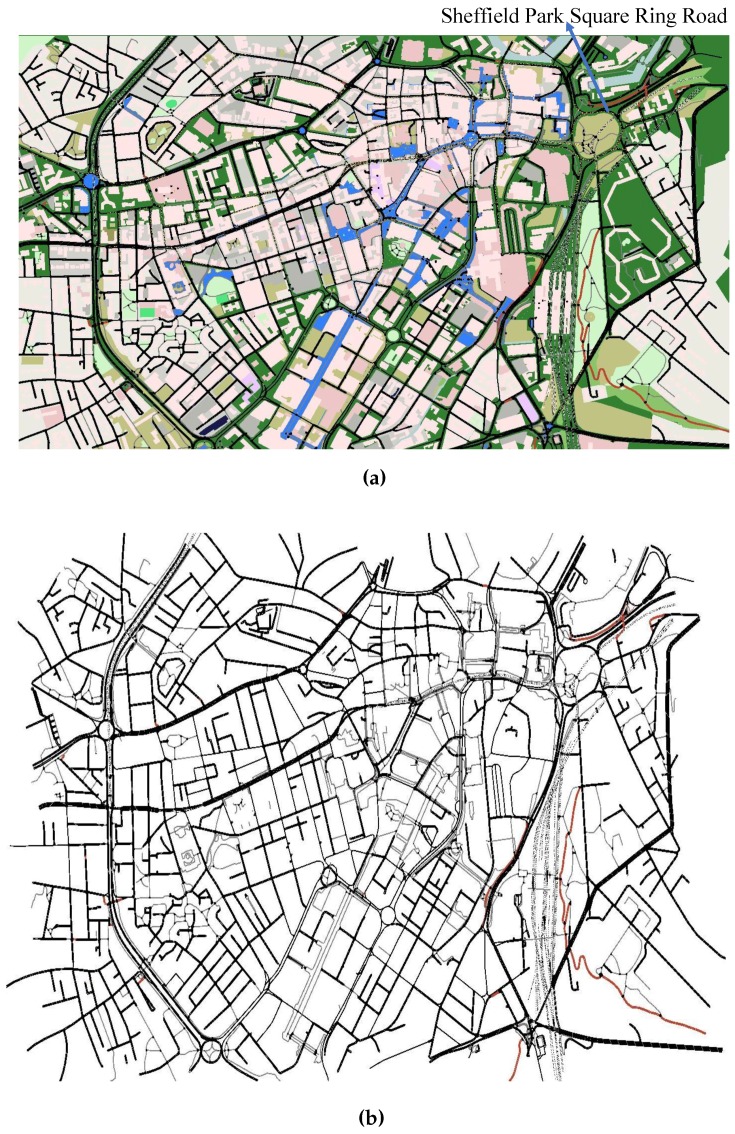
The city centre of Sheffield and the SUMO map. (**a**) The city centre of Sheffield; (**b**) SUMO map of Sheffield city centre.

**Figure 6:**
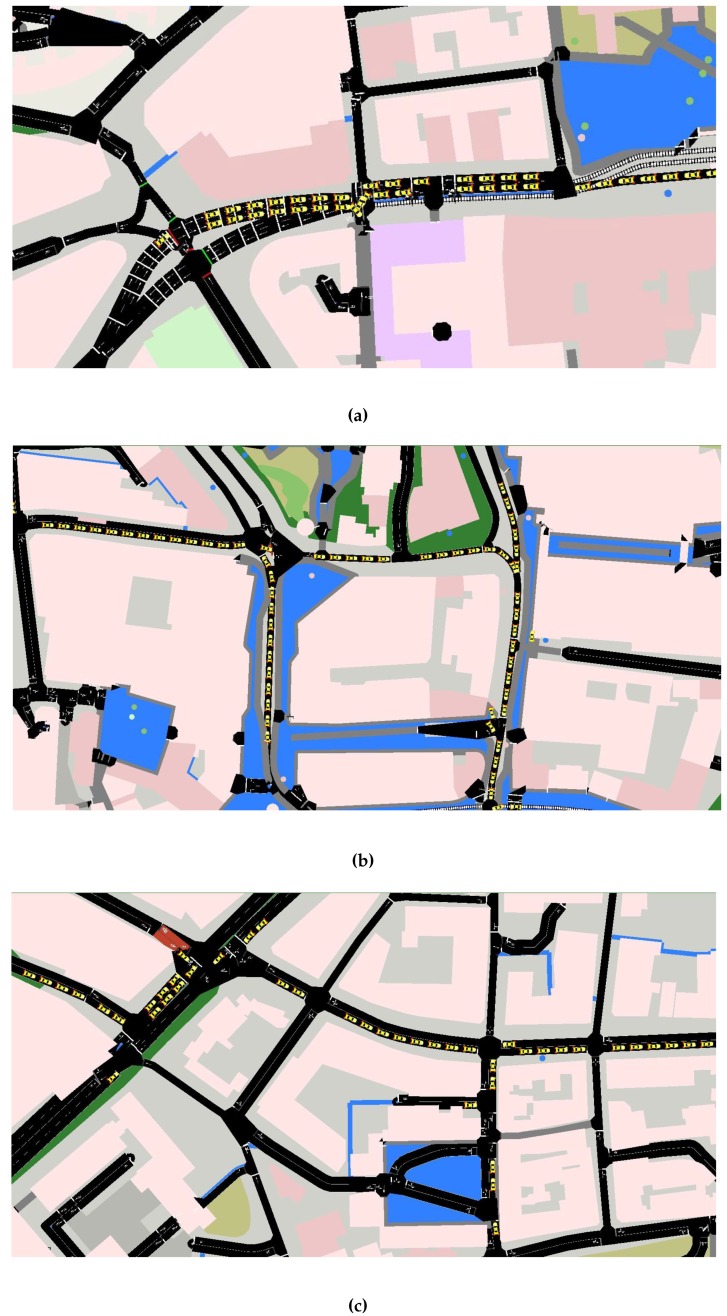
The zoomed places showing traffic congestion on some roads. (**a**) Traffic Congestion Area 1; (**b**) Traffic Congestion Area 2; (**c**) Traffic Congestion Area 3.

The proposed system is simulated using open source software, OpenStreetMap (OSM) [29], to import a real-time map of Sheffield city centre, as shown in Figure 5a,b with and with out parks, lakes and buildings. The congested roads are zoomed in on in Figure 6c,d,e, respectively.

For the first test scenario, we have chosen the city centre of Sheffield since this is a typical urban environment, which contains a variety of roads with different characteristics. For example, there are single-lane roads, the dual carriageway ring road and junctions with restricted access/egress. The level of congestion on these roads varies both geographically and with time. This relationship between congestion and location/time is also present in all major cities. As a result, we think it is reasonable to suggest that the results from Sheffield in terms of the relative performance of each method can be generalized to other cities, as well. The only difference will be that the absolute value, such as mean trip time, may change depending on the size of the city considered.

Table 1 shows the parameters that have been used in the simulation, whereas the vehicles speed and velocity threshold parameters have been chosen by the designer using U.K. road laws as a guide. The other parameters have been chosen based on the OpenStreetMap and SUMO specification.

Table 2 shows that the parameter of SA has been used in this simulation of both the off-line and on-line computation, whereas the values of *T* and *α* are used for the proposed SA-based approach. When making these selections, the following considerations had to be made:

1. A large initial temperature *T* allows for an exhaustive search, but leads to a large computation time. Reducing this initial value will reduce the computation time required at the expense of making it less likely that the globally optimal solution will be achieved.
2. As the value of *α* controls the rate at which Tdecreases, a larger value gives a quicker decrease. This results in a shorter computation. However, this will also result in the algorithm running for fewer iterations, making it less likely to reach the truly optimal solution.

We suggest the values of *T* and *α* given in Table 1 to give a suitable trade-off between the two performance measures considered. Note, there are different values for the off-line and on-line cases, as having a shorter computational time is more desirable for the on-line case than the off-line case, as a real-time implementation would be required. The EMITmodel [30] has been employed, which is a simple statical model of consumption and vehicle emissions based on vehicle speeds and accelerations in the SUMO simulator. In this work, the fuel consumption and *CO*_2_ emissions have been computed based on parameters that have been considered in the cost function (vehicle speed and road length).

The proposed algorithms have been implemented for the different vehicular environments to optimize the traffic scenario. The SAWS optimized the average travel time taken by vehicles to reach their destination, whereas ISATOPSIS has improved most of the criteria (the travel time, fuel consumption and *CO*_2_ emissions) that are used in this paper. The obtained result of the proposed method has been compared to the SAWS, SATOPSIS and DA algorithms.

We initially imported the Sheffield city centre from an OpenStreetMap tool and converted it into the SUMO simulator using the “Netcovert” command. Ten independent Monte Carlo simulations were conducted, and the mean results reported.

The objective of the ISATOPSIS algorithm is to optimize the traffic flow (minimize the travel time, fuel consumption and *CO*_2_ emission). The ISATOPSIS combines the SA algorithm and TOPSIS method as a cost function to optimize different conflicting criteria, such as the length and the average speed. It has successfully minimized the average travel time, fuel consumption and *CO*_2_ emission. However, this has led to a slightly increased average travel distance that has not affected the overall traffic efficiency.

Four different matrices have been measured in the performance evaluation:

- **Mean travel time (MTT)**: the average travel time of all vehicles.
- **Mean travel distance (MTD)**: the average travel distance taken by vehicles.
- **Fuel consumption (FC)**: the average fuel consumption of vehicles.
- **CO2 emission**: the average *CO*_2_ emission of all vehicles.

Table 3 shows the average values of all calculated metrics for all algorithms. This result demonstrates that SAWS minimizes the travel time compared to DA and SATOPSIS. However, it increases the travel distance because it routes the vehicles along the longest free flow paths. The DA has the minimum travel distance comparing to the other algorithms because it routes the vehicles to the shortest path. However, it has the worst performance in terms of travel time, fuel consumption and *CO*_2_ emissions because most vehicles travelling with DA are stuck in congestion. On the other hand, SATOPSIS attempts to minimize all of the matrices by considering multiple attributes in the cost function. It has better performance compared to SAWS, except for the travel time, which converges to some extent with SAWS. In comparison, ISATOPSIS decreases the MTT, FC and *CO*_2_ emissions when compared to DA, SAWS and SATOPSIS. This reduction is due to the re-routing of all vehicles once the congestion is detected. In addition, these results demonstrate the benefits of considering the multiple attribute cost function performed by the ISATOPSIS algorithm to avoid the congestion. However, this re-routing slightly increased the MTD compared to DA and SATOPSIS, respectively. This increase is due to the dynamic re-routing of vehicles, and thus, an extra path has been added to the original route.

Table 4 shows the average (over different vehicle numbers) variances (Var) for the performances measures that have been considered. As the variance values for the proposed method are lower than the comparison methods, this shows that the proposed approach is more consistent than the comparison methods.

**Figure 7:**
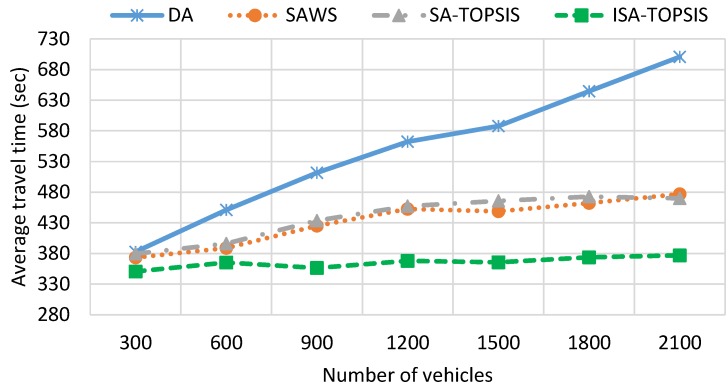
Average travel time.

Figure 7 graphically shows the average travel time of all of the algorithms. It is clear from the figure that the average travel time has a direct relationship with vehicle density. As is foreseeable, the average travel time increases as the number of vehicles increases. This is because of the greater number of vehicles in the traffic jam, which increases the average travel time, as is shown for DA in Figure 7. The SAWS and SATOPSIS travel times remain more constant and lower than the DA. This is due to the distribution of vehicles having the same source/destination over more than one route. In comparison, the ISATOPSIS has significantly improved the average travel time since it re-routes the vehicles to avoid congested roads. In addition, ISATOPSIS pays attention to the congestion, which is not considered in the other algorithms, and attempts to select an optimal path by finding a trade-off between the conflicting objectives.

**Figure 8:**
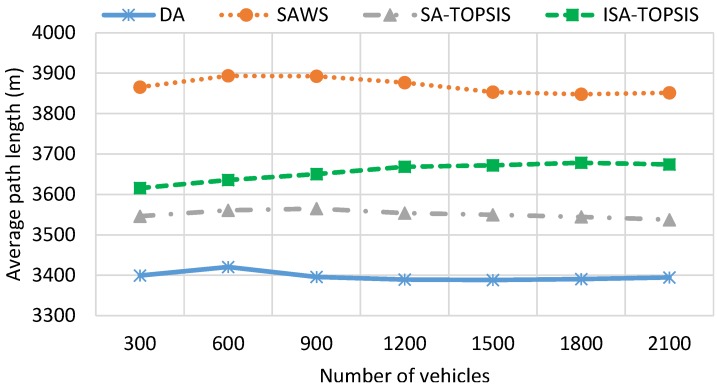
Average travel distance.

Figure 8 illustrates the average path length result for all of the methods considered. The SAWS increases the travel distance compared to DA, SATOPSIS and ISATOPSIS. This is due to the fact that SAWS chooses the paths with the highest average travel speed and distributes the vehicles on them to avoid generating congestion. On the other hand, this result shows that ISATOPSIS can find a compromise by minimizing effectively MTT, FC and *CO*_2_ due to its ability to consider multiple pieces of traffic information. However, this reduction leads to a slight increase in the travel distance compared to DA and SATOPSIS, since ISATOPSIS utilizes the traffic information and re-routes the vehicles to avoid the congested roads, where DA and SATOPSIS have a constant travel distance that is not affected when congestion occurs.

**Figure 9:**
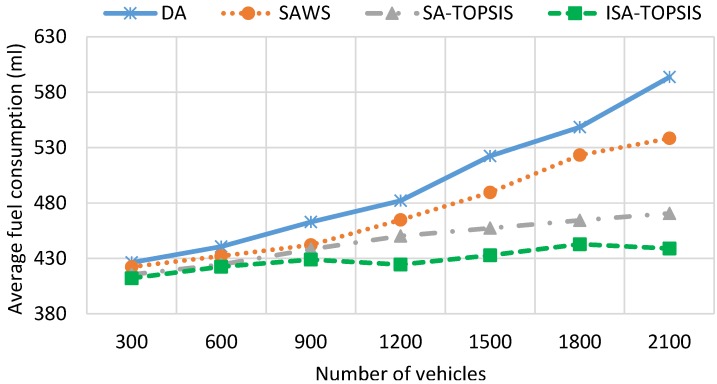
Average fuel consumption.

Figure 9 shows the fuel consumption results obtained by the four algorithms. We can see the impact of taking the longest free flow path and the shortest congested route on the traffic efficiency and the fuel consumption. The fuel consumption result is directly related to the travel time, travel speed, waiting time and travel distance. The highest average speed, the longest travel distance and the most waiting time leads to higher fuel consumption. The figure shows that DA consumes as much fuel as SAWS for low vehicle densities. This is due to the effect of choosing the longest travelled path and waiting times taken by SAWS and DA, respectively. However, with increasing numbers of the vehicles on the city roads, the figure shows that the SAWS fuel consumption is much better than the DA algorithm. This is due to the fact that the longest waiting time is taken by vehicles using DA in the congested area. According to this figure, SATOPSIS and ISATOPSIS consume less fuel when compared to the others. ISATOPSIS has better fuel consumption due to less waiting time, the best average speed and an optimal path that is selected based on the different navigation criteria. In addition, ISATOPSIS pays attention to the congestion with the avoidance mechanism that helps to re-route the vehicles and avoid the traffic jams.

**Figure 10:**
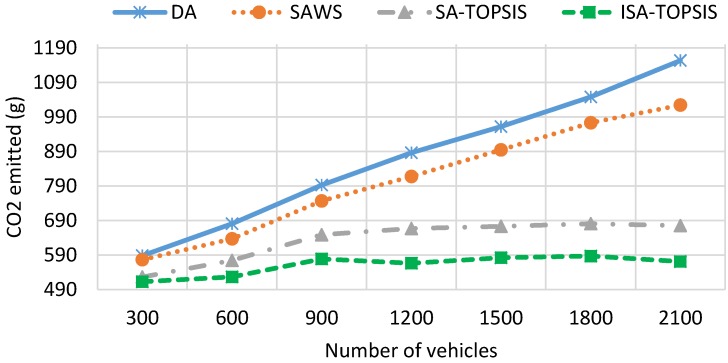
Average *CO*_2_ emission.

Figure 10 depicts the *CO*_2_ emissions recorded from all of the algorithms. The results of *CO*_2_ emissions are directly related to the results of fuel consumption. The longer travel distance, the larger waiting time and the more fuel consumed by the engine result in higher *CO*_2_ emissions. High vehicle densities or traffic congestion lead to longer waiting times on the roads, so the fuel consumption, as well as *CO*_2_ emissions are increased. It is clear from the figure that ISATOPSIS has the lowest average *CO*_2_ emissions compared to the other algorithms. This is due to it having the best average travel speed and the optimal path (multi-attribute cost function) being obtained by ISATOPSIS. The SATOPSIS comes in second place in terms of *CO*_2_ emissions compared to SAWS and DA. Both SAWS and DA have the worst *CO*_2_ emissions due to a large amount of fuel consumed by the vehicles using them.

**Figure 11:**
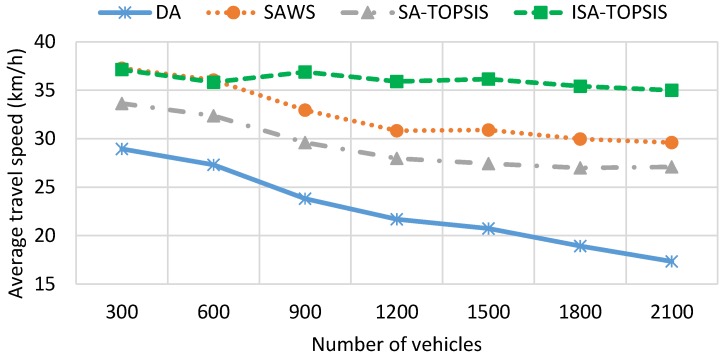
Average travel speed.

Figure 11 illustrates the average travel speed obtained by all of the algorithms. ISATOPSIS has recorded the best average travel speed compared to the other methods at all vehicle densities. This is due to the congestion avoidance mechanism and providing the vehicles with alternative paths to avoid the congested roads. DA has the worst average travel speed. This due to a large number of vehicles being stuck in traffic congestion. We can see the impact of travel speed on the traffic efficiency (see Figure 7 and Figure 8), despite the SAWS and SATOPSIS having better performance comparing to DA. However, they have a relatively poor efficiency compared to ISATOPSIS, due to not paying attention to the congestion avoidance mechanism when traffic jams occur.

Combining all of the results, it is deduced that by using ISATOPSIS and considering multiple pieces of traffic information, the trip time, the fuel consumption, as well as *CO*_2_ emissions of vehicles are optimized, in order to reach the destination via the optimal path.

### 4.2. Scenario of Birmingham City

**Figure 12:**
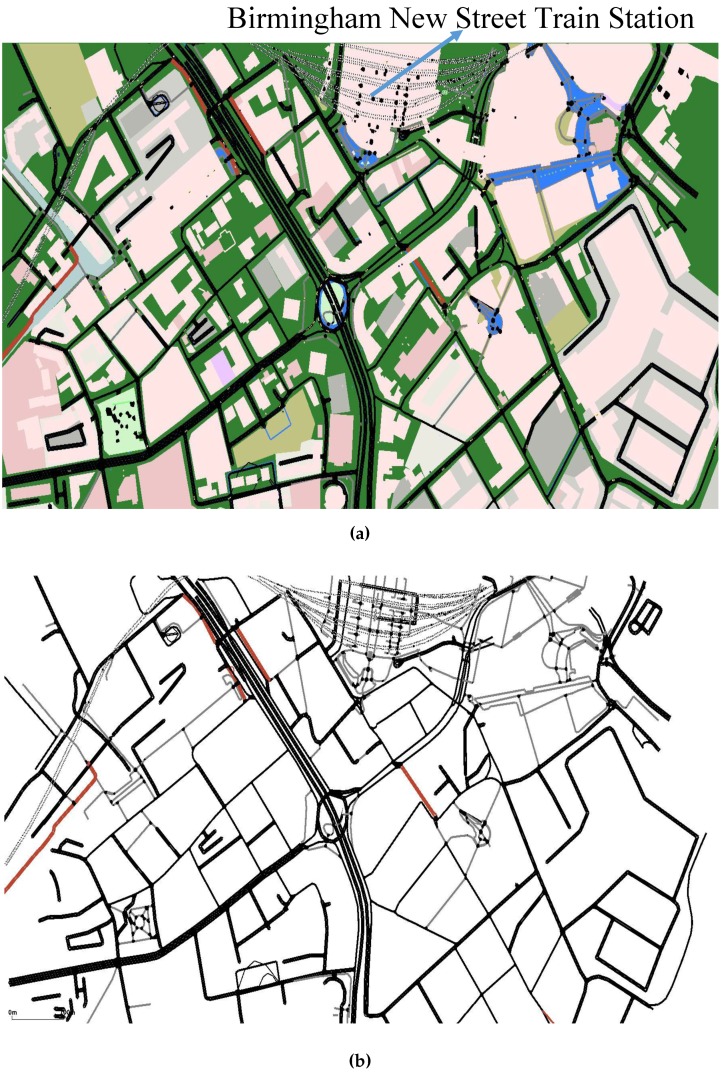
The section of Birmingham city centre and the SUMO map. (**a**) The section of Birmingham city centre under consideration; (**b**) The SUMO section of Birmingham city centre under consideration.

For the second test scenario, a small section of Birmingham city centre has been imported using OSM. Figure 12a,b, respectively, shows the imported area in the SUMO simulator with and without parks, lakes and buildings.

Table 5 shows the parameters that have been used in this scenario, where the number of vehicles has been decreased due to the smaller map size considered in this scenario. Vehicle speed and velocity threshold parameters have been chosen by the designer, again using U.K. road laws as a guide. The other parameters have been chosen based on the OpenStreetMap and SUMO specification. The parameters for the SA algorithm are the same as have been used for the Sheffield test scenario and are summarized in Table 1. The ISATOPSIS, SATOPSIS, SAWS and DA algorithms have also been tested for this scenario to allow further comparisons to be made.

**Figure 13:**
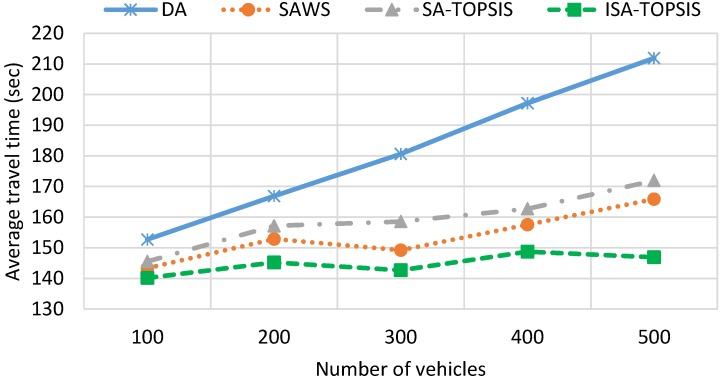
Average travel time in the Birmingham scenario.

**Figure 14:**
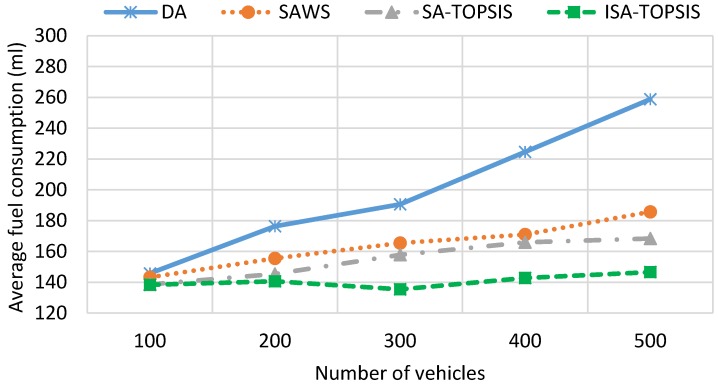
Average fuel consumption in the Birmingham scenario.

Figure 13 and Figure 14 show the mean travel time and fuel consumption results obtained using all of the algorithms being considered. They show that a similar performance pattern has been achieved as was for the previous scenario. Moreover, they show that the ISATOPSIS algorithm still has the best performance as compared to the other algorithms. In conclusion, the relative performances of the methods have not been changed by changing the city under consideration. However, the absolute values of the mean trip times and fuel consumption levels have changed due to the size difference in the maps used. The improved performance over the comparison methods is due to the fact that real-time traffic information has been used to continuously optimize trip time, fuel consumption and *CO*_2_ emissions.

## 5. Conclusions

In this paper, we propose the ISATOPSIS method to address the traffic congestion problem in smart cities. The novelty of this work is in the use of the multi-objective cost function and dynamic route planning. Our proposed method can lead to a reduction in travel time, fuel consumption and *CO*_2_ emissions. The proposed method has been implemented and tested using an OpenStreetMap and the SUMO simulator. Results from the Sheffield scenario show that the simulated annealing weighted sum method can reduce the travel time by an overall average of 19.93% compared to DA and SATOPSIS. This is due to choosing the path with the highest average speed. However, it has a worse performance compared to ISATOPSIS. Simulation results show that our proposed ISATOPSIS method can successfully find a trade-off between different navigation attributes, in order to provide each driver with the least congested path according to the road condition. As reported from the Sheffield test scenario, it is shown that ISATOPSIS can improve the traffic flow by an overall average of 19.22% in terms of travel time, fuel consumption and *CO*_2_ emissions when compared to the Dijkstra, simulated annealing weighted sum and SATOPSIS algorithms. Moreover, similar performance patterns were achieved for the Birmingham-based simulation. In future work, we envisage the route selections being communicated back to intelligent traffic light controls to help adaptively control their sequences to aid in achieving the overall optimal traffic flow for a smart city.

## Figures and Tables

**Table 1:** Simulation parameters as configured in the SUMO implementation of Sheffield scenario.

Simulation Parameters	Value
**Map dimension**	4 km × 3.5 km
**Simulation time**	2500 sec
**Vehicle speed**	0–15 m/s
**Velocity threshold**	7 m/s
**MAC/PHY**	IEEE 802.11p
**Vehicle density**	300–2100 Vehicle
**Route generator**	SUMO

**Table 2:** The tuned SA algorithm parameters of off-line and on-line search.

Parameters	Values
***T* off-line**	500 °C
***α* off-line**	0.998
***T* on-line**	25 °C
***α* on-line**	0.992

**Table 3:** The average results obtained by the Dijkstra algorithm (DA), simulated annealing weighted sum (SAWS), the simulated annealing technique for order preference by similarity to the ideal solution (SATOPSIS) and the improved simulated annealing technique for order preference by similarity to the ideal solution (ISATOPSIS) in the tested scenarios. MTT, mean travel time; MTD, mean travel distance; FC, fuel consumption.

Method	MTT (s)	MTD (m)	FC (mL)	CO_2_ (g)
**DA**	544.45	3396.84	496.603	873.206
**SAWS**	432.55	3868.76	473.194	809.957
**SATOPSIS**	439.29	3551.15	445.629	635.079
**ISATOPSIS**	365.153	3656.367	428.904	560.668

**Table 4:** The overall average variance (Var) results obtained by all algorithms in the tested scenarios.

Method	Var MTT (s)	Var MTD (m)	Var FC (mL)	Var CO_2_ (g)
**DA**	88.202	248.59	96.83	85.47
**SAWS**	65.65	185.819	73.61	61.77
**SATOPSIS**	44.157	136.46	67.408	39.0625
**ISATOPSIS**	26.86	88.25	60.79	32.49

**Table 5:** The simulation parameters configured in the SUMO of Birmingham city.

Simulation Parameters	Value
**map dimension**	2 km × 1.5 km
**Simulation time**	1000 sec
**Vehicle speed**	0–15 m/s
**Velocity threshold**	7 m/s
**MAC/PHY**	IEEE 802.11p
**Vehicle density**	100–500
**Route generator**	SUMO
